# COVID-19 related poor mental health and sleep disorders in rheumatic patients: a citizen science project

**DOI:** 10.1186/s12888-021-03389-7

**Published:** 2021-08-03

**Authors:** Francesca Ingegnoli, Massimiliano Buoli, Cristina Posio, Raffaele Di Taranto, Alessandro Lo Muscio, Enrico Cumbo, Silvia Ostuzzi, Roberto Caporali

**Affiliations:** 1 Division of Clinical Rheumatology, ASST Pini, Milan, Italy; 2 Department of Clinical Sciences & Community Health, Research Center for Adult and Pediatric Rheumatic Diseases, Università degli Studi di Milano, Piazza Cardinal Ferrari 1, 20122 Milan, Italy; 3 Department of Neurosciences and Mental Health, Fondazione IRCCS Ca’Granda Ospedale Maggiore Policlinico, Milan, Italy; 4 Department of Pathophysiology and Transplantation, Università degli Studi di Milano, Milan, Italy; 5 ALOMAR Lombard Association for Rheumatic Diseases, Milan, Italy

**Keywords:** Rheumatic diseases, Perceived stress, Post-traumatic stress disorders, COVID-19, Anxiety, Depressive symptoms, Quarantine, Inflammatory arthritis, Connective tissue diseases

## Abstract

**Background:**

Patients with chronic rheumatic diseases (RDs) are more vulnerable and the containment measures related to the COVID-19 pandemic might have severe psychological consequences. We investigated the presence of and risk factors associated with poor mental health, sleep disorders among RDs during the pandemic.

**Methods:**

This cross-sectional Italian citizen science project evaluated the psychological impact of the COVID-19 pandemic in patients with RDs. Between May and September 2020, eleven RD patients’ associations sent the survey by using their mailing list and the related webpage and social network. 507 RD patients completed an ad-hoc anonymous online survey including the Perceived Stress Scale (PSS) and Impact Event Scale-Revised (IES-R).

**Results:**

The mean scores on the PSS-10 and the IES-R were 18.1 and 29.7, respectively. Higher PSS scores were associated with younger age (*p* <  0.01), female gender (*p* <  0.01), overweight/obesity (*p* = 0.01), psychiatric pharmacotherapy (*p* <  0.01), and anxiety for loss of income (*p* <  0.01). Higher IES-R scores were associated with female gender (*p* <  0.01), intestinal diseases (*p* = 0.03), anxiety (*p* <  0.01), and health concern (*p* <  0.01). Among 375 patients with inflammatory arthritis, 246 (65.6%) had trouble staying asleep, 238 (63.5%) falling asleep, and 112 (29.9%) had dreams about the pandemic. Older age (OR = 1.038, CI 1.002–1.076), psychiatric pharmacotherapy (OR = 25.819, CI 11.465–58.143), and COVID infection (OR = 2.783, CI 1.215–6.372) were predictive of insomnia during the pandemic.

**Conclusions:**

A considerable COVID-19 related psychosocial burden has been detected in RDs. Different factors were predictive of poor mental health and sleep disorders in these patients. Focused supportive strategies should be implemented to improve the psychological well-being of fragile patients during pandemics.

**Supplementary Information:**

The online version contains supplementary material available at 10.1186/s12888-021-03389-7.

## Background

The coronavirus disease 2019 (COVID-19) pandemic has spread rapidly worldwide. In early 2020, the main European epicenter was in Lombardy (Italy) with more than 600,000 confirmed cases and 30,600 deaths to date, on nearly 10,000.000 inhabitants. Italian Government had to apply unprecedented mitigation policies proclaiming a national lockdown from 9th March to 4th May, leading to significant social and lifestyle changes that affected nearly every aspect of daily life.

Moreover, during the first wave of the pandemic, the healthcare system was overwhelmed by the ongoing outbreak of COVID-19, and this emergence led to unprecedented changes in the clinical management of chronic patients with rheumatic diseases (RDs) with many cancellations or delayed of routine medical appointments [1–5]. RDs are chronic inflammatory disease and RD patients are more vulnerable and sleep disturbance, pain and mood appear to be linked to dysfunctions in circadian rhythms [6–8]; indeed, disease activity, increased pain, fatigue, and psychological factors such as depression and anxiety may negatively affect daily-life activities leading to sleep disorders [9–11]. This latter has a key role in the vicious circle in maintaining chronic pain, mood symptoms, fatigue, and functional impairments creating a dysfunctional cascade characterized by all the major concerns reported by patients with RDs [11–14].

Additionally, a short- and long-term psychosocial burden is one of the relevant consequences of the COVID-19 pandemic [15, 16]. Based on lockdown experiences that were recorded in response to previous epidemics, quarantine measures might have negative psychological effects, including symptoms of post-traumatic stress disorders (PTSD), stress, anxiety, and depression [17–19]. Besides, decreased levels of physical activity and exposure to daylight, as well as changes in routines and mental health concerns, have led to increased incidence of sleep disorders [20]. At present, only scattered data on the psychological impact of the pandemic on Dutch and Turkish patients with RD are available. These studies used different scales to assess the pandemic impact and are not comparable. Thus, the real burden and risk factors of COVID-19 related mental health symptoms on Italian rheumatology patients following the peak of the outbreak is still unknown.

The present study addresses the lack of information on the relationship between COVID-19 and mental health symptoms, sleep disorders and to identify potential factors associated with these concerns. To our knowledge, this study is the first Italian nationwide citizen science project with the active and voluntary participation of associations of patients with chronic rheumatic conditions. The results will be useful to identify RD patients at risk to develop psychiatric symptoms and to implement prevention strategies that can avoid poor mental health in these subjects.

## Methods

### Study design

The current citizen science cross-sectional study was conducted online to evaluate the psychological impact of the COVID-19 pandemic in Italian patients with RDs.

A structured meeting between a patient representative from the Lombardy Association of patients with RDs (ALOMAR) and medical specialists (rheumatologist and psychiatrist) was convened to discuss the psychological burden related to the pandemic in RDs. Based on support requests received by the patients’ association, this online survey called INSIEME, meaning “together” in English was designed. The ethical committee of the University of Milan approved this study (07.05.20–47/20). The survey was for adult patients with RDs (inclusion criteria); it was anonymous, and information could not be verified.

The survey was composed of three parts (supplementary materials). Firstly, participants were all explicitly asked if they were willing to complete the survey and they were informed that their consent would permit them to evaluate the psychological impact of the current pandemic. Then, patients were also asked to self-report demographic, disease characteristics, comorbidities, COVID-19 infection (confirmed by nasal-pharyngeal swab, probable, absent), and their major sources of anxiety. Participants also were asked to indicate the presence of anxiety, depression, or sleep disturbance and related treatment before and after the lockdown period.

In the second part, RD patients were asked to complete the 10-item Perceived Stress Scale (PSS) [21] [22, 23] and in the last section the 22-item Impact of Event Scale-Revised (IES-R).

### Perceived stress scale

The 10-item PSS [21] is a validated self-administered questionnaire widely used to assess stress perception during the previous month. Responses to each question are categorized on a 5-point Likert scale from 0 (never) to 4 (very often). The positively worded items of the PSS-10 (4, 5, 7, and 8) were reverse scored. The total scores range from 0 (no stress) to 40 (high stress). Thresholds used in the literature consider the stress level low for scores between 0 and 13; moderate, between 14 and 26; and high, greater than 26 [22, 23].

### Impact of event scale-revised

The 22-item IES-R is a screening measure used to measure the individual response to a specific traumatic event. It has three subscales (intrusion, avoidance, and hyperarousal), and a total subjective stress score. Participants rate the extent to which each item applies to their experiences during the preceding 7 days, from 0 to 4. The total score ranges from 0 to 88. The threshold established in the literature is the following: a total score ≥ of 33 indicates the probable presence of PTSD [24]. Moreover, we considered items about sleep quality: question 2 (trouble staying asleep), question 15 (trouble falling asleep), and question 20 (dreams about it).

### Patient involvement and data collection

A call for this web-based survey completion was sent using the ALOMAR mailing list and the related webpage and social network, and eleven National patients’ associations of RDs among which the National Association of People with RDs (ANMAR) and National Association of People with Rheumatologic and Rare Diseases (APMARR) contributed to the survey dissemination.

Completion was voluntary, anonymous and participants were not remunerated. The measurement interval took place between May and September 2020, after the first pandemic wave. It was conducted using an internet-based program supported for data protection by the IT service of the Università degli Studi di Milano (UNIMI). The IT service also collected all data and provided a database for the analysis.

The way of collecting data with the dissemination of the questionnaire through social networks and the creation of a specific platform place this project in the field of citizen science studies.

### Statistical analysis

The results of the study were summarized using absolute numbers and percentages and reported according to the GRIPP2 checklist [25, 26].

Results were summarized by descriptive statistics using mean, standard deviations, absolute numbers, and percentages. All replies were mandatory, the majority of fields were checkboxes or dropdowns to limit inaccurancies, no missing data were present. Due to the method of dissemination (mailing list, social network, and website), the response rate cannot be determined. Based on the respondents’ diagnosis, patients were stratified according to three main groups of RDs (i.e. inflammatory arthritis, connective tissue disease –CTDs-, and primary fibromyalgia). Five patients were excluded, as their diagnosis did not fit the above-mentioned groups.

A linear regression analysis was firstly performed to verify the association between IES-R and PSS total scores. PSS and IES-R total score was compared between groups defined by qualitative variables through one-way analyses of variance (ANOVA) with Bonferroni’s posthoc analyses in case of three or more groups; correlation analyses (Pearson’s correlation) were performed to analyze the relation between PSS total score and quantitative variables. Subsequently, three linear multivariable regression models for each rating scale were performed considering the statistical significance of the previous analysis: 1) demographic and clinical variables: diagnostic group, age, gender, Lombardy as a region of residency yes/no, disease duration; 2) medical comorbidity variables: COVID-19 symptoms yes/no, hypertension, gastritis, bowel diseases, overweight/obesity; 3) mental health variables: sources of anxiety, depressive symptoms, assumption of medication for psychiatric symptoms before COVID-19, presence of anxiety, current prescription of psychiatric drugs, prescription of psychiatric therapies before COVID-19. These factors were the independent variables while PSS and IES-R scores were the dependent ones. The variables that resulted statistically significant in these three models were inserted in a further final multivariate regression model. The validity of all models was verified by the Durbin-Watson test. The level of statistical significance for all the statistical analyses was set at *p* ≤ 0.05.

Concerning sleep disorders, we chose to analyze only the group of patients with inflammatory arthritis as more homogeneous and larger. According to IES-R item (*2–15-20*) scores, we considered poor sleepers those who reported scores ≥1; moreover, according to patients’ answers, we created a new qualitative variable about the presence of sleep disturbances during the pandemic (Yes or No). The two groups identified by this variable were compared by independent-sample t-tests about quantitative variables, while qualitative variables were compared by chi-square tests [χ2]. A binary logistic regression model was then performed considering the presence or absence of sleep disorders during the pandemic as a dependent variable and statistically significant variables in the univariate analyses (t and χ2 tests) as independent ones. All analyses were performed using SPSS version 26.

## Results

### Sample characteristics

In total, 507 RD patients completed the survey (Table 1). 375 (73.9%) patients had inflammatory arthritis (243 rheumatoid arthritis, 76 psoriatic arthritis, 49 ankylosing spondylitis, and 7 Still’s disease). 96 (18.9%) patients had CTDs or systemic vasculitis (22 undifferentiated CTDs, 6 mixed CTDs, 1 polymyositis, 27 systemic sclerosis, 10 Sjögren’s syndrome, 23 systemic lupus erythematosus, 5 vasculitis, 2 primary antiphospholipid syndromes). 31 (6.1%) patients had primary fibromyalgia and 5 osteoarthritis or crystal arthropathies. The RD population was mainly composed of women (417 [82.3%]), the median (IQR) age was 54 (44–63) years and the median (IQR) disease duration was 10 (5–20) years. Results were obtained from survey responses; no medical records were reviewed. The most frequent comorbidity was gastroesophageal reflux disease, reported by 85 (16.8%) of patients (Table 1).

**Table 1:** Characteristics of 507 respondents

	Total (*n* = 507)
**Gender** Female n (%)	417 (82.2%)
**Age** yrs., median (Q1, Q3)	54 (44–63)
**Disease duration** yrs., median (Q1, Q3)	10 (5–20)
**Diagnosis**, n (%)	
inflammatory arthritis	375 (73.9%)
connective tissue diseases	96 (18.9%)
primary fibromyalgia	31 (6.1%)
miscellaneous	6 (1.1%)
**Comorbidities**, n (%)	
Arterial hypertension	95 (18.7%)
Diabetes	17 (3.4%)
Cardiovascular disease	16 (3.2%)
Overweight/obesity	54 (10.7%)
Gastritis	45 (8.9%)
Gastroesophageal reflux disease	85 (16.8%)
Intestinal diseases	36 (7.1%)
Thyroiditis	70 (13.8%)
Ocular diseases	42 (8.2%)
**Resident in Lombardy** n (%)	412 (81.3%)
**COVID-19 Infection** n (%)	63 (12.4%)

Self-reported characteristics of 507 respondents with rheumatic diseases

Among the participants, 412 (81.3%) lived in Lombardy, the region with the highest rates of infection. 63 (12.4%) reported an infection of COVID-19 confirmed by positive laboratory test and/or swab or probable (i.e. symptoms compatible with COVID-19).

### The severity of mental health outcomes and associated factors

**Fig. 1:**
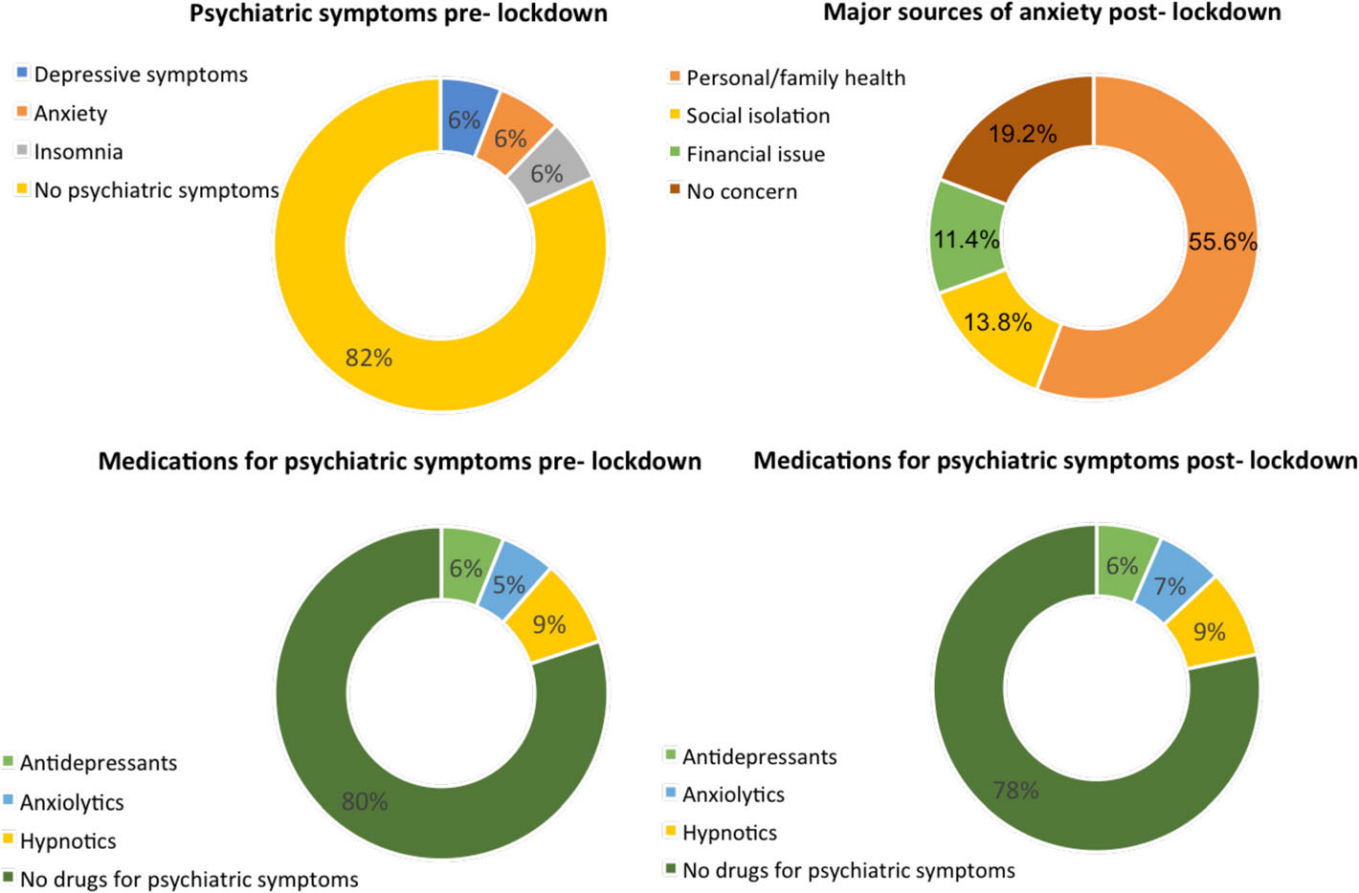
Self-reported major sources of anxiety pre- and post-lockdown period among 507 Italian patients with rheumatic diseases

A small proportion of participants reported psychiatric symptoms before pandemic: depressive symptoms (30, 5.9%), anxiety (32, 6.3%), and insomnia (31, 6.1%). The self-reported use of medication for these symptoms increased from pre- to post-lockdown period: antidepressants (31 [6.1%] vs 33 [6.5%]), anxiolytics (27 [5.3%] vs 33 [6.5%]) and hypnotics (43 [8.5%] vs 44 [8.7%]). As shown in Fig. 1, the major sources of anxiety were related to personal and relatives’ health (282 [55.6%]), social isolation (70 [13.8%]), and financial issues (58 [11.4%]).

The mean (SD) scores on the PSS-10 for perceived stress and the IES-R for distress for all respondents were 18.1 ± 8.1 and 29.7 ± 17.5, respectively. The scores of the two rating scales were significantly associated (β = 0.65. *P* <  0.01). Concerning the IES-R subscale scores, the total sample did not show the prominence of one of the three main domains (intrusion, avoidance, and hyperarousal). Patients with fibromyalgia had higher scores in all scales, and subscales compared with other rheumatic diseases (Table 2).

**Table 2:** Total scores and severity categories of rating scale scores according to diagnostic groups

	Inflammatory arthritis (*n* = 375)	CTDs/vasculitis (*n* = 96)	Primary fibromyalgia (*n* = 31)	Total (*n* = 502)*
**IES-R score**^**Δ**^				
Total, mean ± SD	28.7 ± 17.7	30.5 ± 17.3	38.0 ± 16.1	29.7 ± 17.5
Normal, n (%)	234 (62.4%)	51 (53.1%)	11 (35.5%)	296 (59.0%)
Probable PTSD, n (%)	141 (37.6%)	45 (46.9%)	20 (64.5%)	206 (41.0%)
**IES-R Subscale** mean ± SD				
intrusion	10.03 ± 7.20	11.26 ± 7.23	12.77 ± 7.02	10.4 ± 7.2
avoidance	10.24 ± 6.31	10.25 ± 5.84	13.06 ± 6.27	10.4 ± 6.2
hyperarousal	8.48 ± 5.94	8.98 ± 6.15	12.23 ± 5.38	8.8 ± 5.9
**PSS-10 score**^**◊**^				
Total, mean ± SD	17.4 ± 8.3	19.8 ± 7.6	21.6 ± 6.5	18.1 ± 8.1
Low (≤ 13), n (%)	126 (33.6%)	23 (24.0%)	3 (9.7%)	152 (30.3%)
Moderate (14–26) n (%)	184 (49.1%)	58 (60.4%)	19 (61.3%)	261 (52.0%)
High (≥27) n (%)	65 (17.3%)	15 (15.6%)	9 (29.0%)	89 (17.7%)

*5 of 507 patients had a diagnosis that did not fit with any of the diagnostic groups and were excluded from the analysis

^Δ^ IES-R score ≥ 33 is indicative of probable Post Traumatic Stress Disorder (PTSD). Difference between diagnostic groups: Χ^2^ = 10.25, df = 2, *p* = 0.006

◊ Difference between diagnostic groups: Χ^2^ = 11.93, df = 4, *p* = 0.02

Total scores and severity categories of rating scale scores according to the main rheumatic diagnostic groups

### Factors associated with mental health outcomes

The final multivariate regression model showed that independent variables associated with a higher PSS-10 total score were: female gender (β = 0.12, *P* <  0.01), younger age (β = − 0.23, *P* <  0.01), residence outside Lombardy (β = − 0.09, *P* = 0.03), presence of overweight/obesity (β = 0.11, *P* = 0.01), on-going therapy with psychiatric compounds (β = 0.26, *P* <  0.01) and anxiety related to loss of incomes (β = 0.19, *P* <  0.01), see Table 3.

**Table 3:** Factors significantly associated with mental health outcomes (at least one of the two rating scales) identified by multivariate regression analysis

	PSS-10		IES-R	
	***β***	***p***	***β***	***p***
Gender*	0.12	**< 0.01**	0.14	**< 0.01**
Age	−0.23	**< 0.01**	−0.09	0.06.
Region of residence^Δ^	−0.09	**0.03**	−0.10	**0.02**
***Comorbidities***				
Overweight/obesity^◊^	0.11	**0.01**	0.07	0.12.
Intestinal diseases^◊^	0.05	0.19	0.10	**0.03**
Anxiety disorder^◊^	0.08	0.07.	0.19	**< 0.01**
***After lockdown***				
Psychopharmacology^◊^	0.26	**< 0.01**	0.12	0.22.
Sources of anxiety^ω^	0.19	**< 0.01**	0.13	**< 0.01**

*The variable was codified as female = 2; male = 1

^Δ^ The variable was codified as 0 = living in a region different from Lombardy; 1 = living in Lombardy

^◊^The variable was codified as 0 = no; 1 = yes

^ω^The variable was codified as 0 = no source of anxiety; 1 = health; 2 = work and finances; 3 = social isolation

*β* = standarizedregression coefficient

IES-R: Impact of Event Scale – Revised; PSS-10: Perceived Stress Scale; ns: not significant

In bold statistically significant p

Factors significantly associated with mental health outcomes (at least one of the two rating scales) identified by multivariate regression analysis

Regarding IES-R (Table3), the multivariate regression model highlighted that independent variables associated with a higher IES-R total score were female gender (β = 0.14, *p* <  0.01), living in Lombardy (β = − 0.01, *p* = 0.02), intestinal diseases (β = 0.10, *p* = 0.03), anxiety (β = 0.19, *p* <  0.01) and anxiety related to worries about health (β = 0.13, *p* < 0.01).

### Sleep disorders and insomnia predictors in patients with inflammatory arthritis

**Fig. 2:**
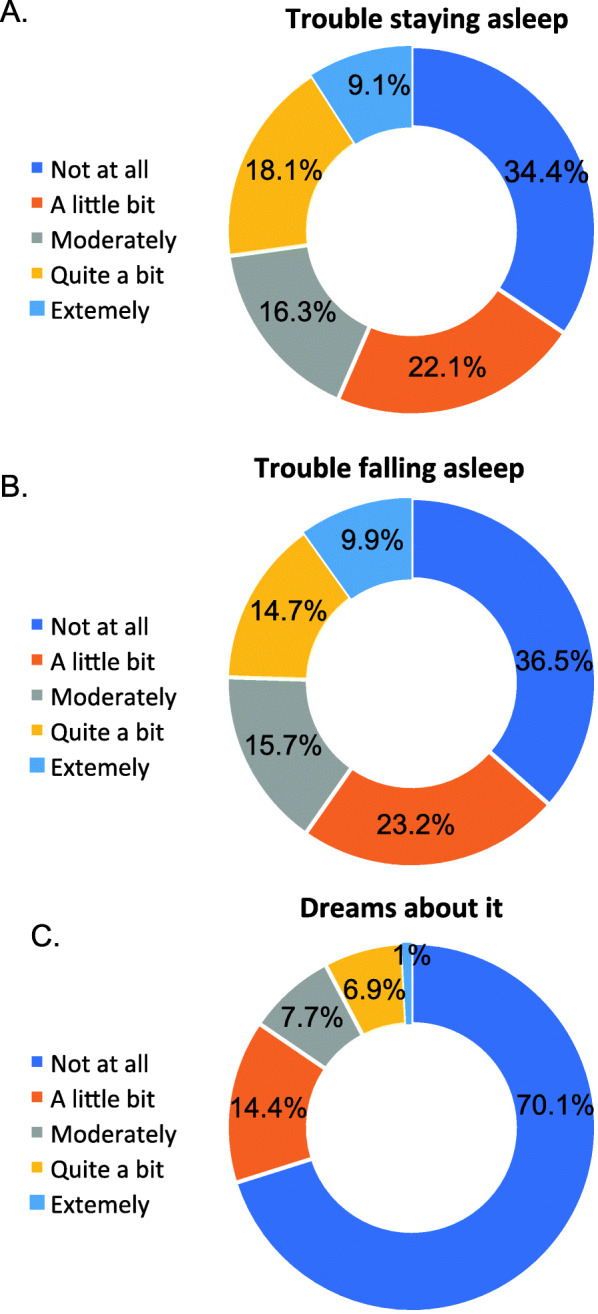
Self-reported sleep disorders after COVID-19 lockdown period by Italian patients with inflammatory arthritis: (**A**) trouble staying asleep, (**B**) trouble falling asleep, and (**C**) dreams about the pandemic

Finally, we focused on patients with inflammatory arthritis. Results of questions about sleep are shown in Fig. 2. Patients reporting insomnia had older age [*t* = 2.844, *p* = .005] and higher PSS total score [*t* = 3.114, *p* = .003]. In addition, patients with insomnia had: more comorbidities (χ^2^ = 7.416, df = 1, *p* = .009), cardiovascular diseases (χ^2^ = 5.721, df = 1, *p* = .039), depressive symptoms (χ^2^ = 2.778, df = 1, *p* = .002), gastritis (χ^2^ = 4.140, df = 1, *p* = .053), bowel diseases (χ^2^ = 6.603, df = 1, *p* = .022), history of treatment for a psychiatric disorder ***(***χ^2^ = 53.907, df = 1, *p* < .001), more medications for psychiatric symptoms before COVID-19 (χ^2^ = 101.446, df = 1, *p* < .001) and a more frequent COVID-19 diagnosis (χ^2^ = 6.284, df = 1, *p* = .018).

The goodness-of-fit test results (Hosmer and Lemeshow Test: χ^2^ = 8.078, df = 8, *p* = .426) showed that the binary logistic regression model was reliable, allowing for a correct classification of 90.7% of the cases. In addition, the model was overall significant (Omnibus test: χ^2^ = 87.068, df = 10, *p* < .001). Age (OR = 1.038, *p* = .040), assumption of medication for psychiatric symptoms before COVID-19 (OR = 25.819, CI 11.465–58.143) and the presence of COVID infection (OR = 2.783, CI 1.215–6.372) resulted to be predictors of insomnia during the pandemic.

## Discussion

This study revealed a consistent psychological burden among rheumatic patients after confinement during the COVID-19 pandemic in Italy in terms of the high impact of self-reported distress, anxiety, perceived stress, and sleep disorders. Among the identified risk factors, having female gender, younger age, living outside Lombardy, having overweight/obesity, or intestinal diseases, having a history of psychiatric symptoms (e.g. anxiety), and having to experience anxiety for financial or health issues were associated with poor mental health.

According to self-reported psychiatric symptoms and related therapy pre- and post-lockdown, we observed a worsening of symptoms and an increase in the assumption of psychiatric drugs in this vulnerable population. Our results are consistent with data reported in other cohorts of rheumatic patients during the initial stage of the COVID-19 epidemic [27, 28]. In addition, the impact of trauma was found to significantly influence the severity of stress perceived by patients in agreement with previous reports [24].

In particular, stress and PTSD were perceived more by females. This is not surprising as females and males react to stressful events differently in terms of coping strategies, psychological and biological mechanisms [29–31]. These results are also confirmed by data on the psychological impact of the COVID-19 pandemic on the Italian population [32–34] and Turkish RDs [27].

Moreover, younger adults were found to have higher levels of stress. This is in line with other Italian studies during the COVID-19 outbreak [32, 33, 35], and also with previous studies highlighting that older adults present greater self-control, emotional self-regulation, and better coping strategies compared to younger adults [36, 37]. A further potential explanation is that younger people might experience higher stress levels because they increased the use of the Internet and social media during the lockdown period [38–40].

The current study found a significant association between overweight/obesity and higher levels of perceived stress, while the presence of intestinal diseases and anxiety disorders were related to PTSD. A recent meta-analysis reported that body mass index is directly associated with perceived stress [41]. Thus, the COVID pandemic might have been perceived as more stressful in the light of poor outcomes associated with the infection in subjects affected by overweight or obesity [42]. Previous studies found PTSD both in inflammatory and functional intestinal diseases [43, 44].

In the context of the COVID-19 pandemic in Italy, the first wave was much more serious in Lombardy than in the other regions. By contrast, our results showed that patients living in regions different from Lombardy had higher PSS-10 and PTSD scores after lockdown. This supports the hypothesis that psychological impact was not only related to direct COVID-19 exposure but also to the media storm that provided a general sense of threat [45]. Furthermore, Lombardy is a highly urbanized region, while in rural areas patients may experience greater difficulty in accessing health facilities in case of emergency [46].

As expected, specific sources of anxiety were related to PSS-10 and PTSD scores. Notably, our data showed that worries about loss of employment and incomes had a greater impact on perceived stress levels after lockdown. These results are consistent with data reported by an Italian study assuming that higher incomes are associated with lower levels of stress [35], while health concern is related to psychological distress. The same sources of anxiety were reported in other studies during a pandemic [47–49]. Moreover, these results are consistent with those during quarantines showing that patients with chronic diseases perceived more stress as access to regular medical care and prescriptions were problematic [17].

In the context of the post lockdown phase of the pandemic, while there was a gradual restoration of outpatient services, PTSD was found in 41% of participants. This appears particularly important for the interpretation of the high rate of severe self-reported distress symptoms. Therefore, it appears to support the concern about the risk of PTSD as the second tsunami of the COVID-19 pandemic [50]. In our data, the main PTSD cluster of symptoms (intrusion, avoidance, and hyperarousal) was balanced without the prominence of none of the domains.

Moreover, sleep disturbances were a relevant concern in patients with inflammatory arthritis, with higher rates of poor sleepers compared to the general Italian population, suggesting that people affected by inflammatory arthritis are more vulnerable to COVID psychological aftermaths [11, 18].

Furthermore, our results showed that older patients who had coronavirus infection and were previously treated for psychiatric disorders were at higher risk of developing sleep disorders. Concerning age, this could be explained by age-related changes in circadian rhythms and consequent higher prevalence of insomnia among older people [51]. The previous use of psychiatric compounds in subjects affected by insomnia during the pandemic is not surprising as sleep disturbances are generally observed in patients affected by mental disorders, particularly depression and anxiety [52]. Besides, complaints such as difficulty falling or staying asleep, unsatisfying sleep, irritability, and nightmares are well documented in some anxiety disorders, such as generalized anxiety disorder and PTSD. Finally, our study confirmed COVID-19 infection to be an important contributing factor to the development of insomnia. Indeed, several studies proved that survivors after SARS-CoV-2 had negative psychosocial aftermaths; notably, it seems that both immune activation towards the virus and pandemic-related stressors (e.g. isolation, concerns about infecting relatives, financial difficulties) can induce detrimental effects on patients’ mental health including poor sleep quality.

It is well established that sleep disruption may worsen arthritis, leading to joint stiffness, pain, weakness, anxiety, depression, and poor outcome [12, 53]. Moreover, impaired sleep may affect work productivity, social functioning, and daily activities, proving to be a considerable psychosocial burden [54].

Some limitations should be considered in the interpretation of these results. First, although the number of respondents is quite large, it represents a part of the RD patients, and self-selection bias may have influenced the results. Second, the cross-sectional design of the study prevents drawing any cause-effect conclusion and the response rate cannot be calculated. Third, although the survey had nationwide dissemination, the respondents were mainly from Lombardy, probably because it was the Italian region most hit during the first wave of the pandemic.

Despite limitations, our findings may have a vested interest for both patients and physicians to support present and post-pandemic interventions related to the COVID-19 pandemic that could be useful for mitigating the psychological impact on more vulnerable patients. Moreover, both stress and PTSD are known triggers for relapse autoimmune diseases; thus, these aspects together with delayed routine medical appointments are inevitably intertwined there is a concern about potential disease flares to which physicians should pay particular attention. Besides, the present findings will be of help to patients’ associations that may implement measures for psychological support to alleviate patient distress around COVID-19.

## Supplementary Information

**Additional file 1.**

## Data Availability

The data that support the findings of this study are available from the corresponding author upon reasonable request.
