# Supplementary material for: COVID-19 related poor mental health and sleep disorders in rheumatic patients: a citizen science project
Source: BMC Psychiatry. 2021 Aug 3;21:385. doi: 10.1186/s12888-021-03389-7 (PMC8330176; doi:10.1186/s12888-021-03389-7)
Supplement: Supplementary file 1 — Additional file 1. [file 12888_2021_3389_MOESM1_ESM.docx]

**SUPPLEMENTARY MATERIALS**

Questions of the online survey

General Information

The questionnaire is for adult patients. The system is not able to prevent the compilation of minors.

**1.** Please indicate your age**:**

**2.** Gender: *Male, Female, other*

**3.** Are you living in Lombardy?

□ *yes*

□ *no*

**3a.** If you do not reside in Lombardy, in which region do you live?

- Lazio
- Campania
- Sicilia
- Veneto
- Emilia-Romagna
- Piemonte
- Puglia
- Toscana
- Calabria
- Sardegna
- Liguria
- Marche
- Abruzzo
- Friuli-Venezia-Giulia
- Trentino-Alto Adige
- Basilicata
- Umbria
- Molise
- Valle d’Aosta

**4.** Please, indicate the rheumatic diseases you are suffering from:

- Psoriatic arthritis
- Rheumatoid arthritis
- Osteoarthritis
- Undifferentiated connective tissue disease
- Mixed connective tissue disease
- Dermatomyositis
- Fibromyalgia
- Gout and concrocalcinosis
- Systemic lupus erythematosus
- Rheumatic polymyalgia
- Polymyositis
- Systemic sclerosis
- Sjögren's syndrome
- Ankylosing spondylitis
- Vasculitis
- Other

**5.** In what year did you have the diagnosis? Consider the most recent rheumatological disease.

□ *year of diagnosis*

**6.** Are you suffering from other non-rheumatological diseases? If so, can you tell us which ones?

no

yes, specify:

- Hypertension

- Diabetes mellitus

- Cardiovascular diseases

- Overweight - Obesity

- Depressive symptoms

- Anxiety

- Gastritis

- Gastroesophageal reflux

- Other intestinal diseases

- Thyroiditis

- Eye diseases

- Other

**7.** Before the outbreak of COVID-19, were you being treated for any of the following symptoms?

- Anxious symptoms

- Depressive symptoms

- Insomnia

- No, I was not being treated for any of these symptoms

**8.** Before the outbreak of COVID-19, were you taking medications for any of the following symptoms?

- Medication for anxiety symptoms

- Medication for depressive symptoms

- Medication for insomnia

- No, I wasn't taking medication for these symptoms

**9.** Did you get sick with COVID-19?

- Yes, with certainty because I had a positive swab and I was hospitalized

- Yes, with certainty because I had a positive swab but I was not hospitalized

- Probably yes, I have had flu-like symptoms but I did not swab

- I haven't had any symptoms

**10.** Are you currently taking medication for any of the following symptoms?

- Medication for anxiety symptoms

- Medication for depressive symptoms

- Medication for insomnia

- No, I'm not taking any medications for these symptoms

**11.** Could you please tell us what were your main sources of anxiety during this period?

- **10-item Perceived Stress Scale**
- **22-item of the Impact of Event Scale-Revised**
